# Pulmonary Function Test Abnormalities in Children with Sickle Cell Anemia: A Cross-Sectional Study from a Tertiary Care Centre in Odisha, India

**DOI:** 10.7759/cureus.77977

**Published:** 2025-01-25

**Authors:** Sanjay Kumar Sahu, Manas Ranjan Behera, Nikhila P Gannavarapu, Palash Das, Amrut Mohapatra, Asish R Mohakud

**Affiliations:** 1 Pediatrics, Kalinga Institute of Medical Sciences, Bhubaneswar, IND; 2 Pulmonary Medicine, Kalinga Institute of Medical Sciences, Bhubaneswar, IND; 3 Pediatric Cardiology, Kalinga Institute of Medical Sciences, Bhubaneswar, IND

**Keywords:** children, fev1/fvc, pulmonary function test, restrictive lung disease, sickle cell anemia

## Abstract

Background: This cross-sectional study analyzed the pulmonary function in children with sickle cell disease (SCD), assessing the pulmonary abnormalities and comparing these with a control group of children with other forms of anemia.

Materials and methodology: This study was conducted from July 2022 to June 2024 at Kalinga Institute of Medical Sciences, Bhubaneswar, Odisha, India, and included 126 children (63 with SCD, 63 with other forms of anemia) aged between six to 18 years. Anthropometric data, clinical history, and laboratory parameters were collected, and pulmonary function tests (PFTs) were performed using spirometry to evaluate forced vital capacity (FVC) and forced expiratory volume in one second (FEV1).

Results: Children with SCD had lower height and weight than those in the control group, with height differences reaching statistical significance. Hemoglobin levels were higher in SCD cases than those in the control group, despite both groups exhibiting anemia. The PFTs revealed lower mean FVC and FEV1 in SCD patients, with a predominant restrictive pattern observed in 40 children with SCD (63.4%) compared to 25 children (39.6%) in controls. Only 17 children with SCD (29.6%) showed normal PFT results. Among risk factors, older age, history of vaso-occlusive crises, acute chest syndrome, and blood transfusions were linked to restrictive abnormalities, though not statistically significant. Children on hydroxyurea showed a trend toward restrictive PFT patterns.

Conclusion: The study underscores the high prevalence of restrictive lung function abnormalities in children with SCD, highlighting the importance of regular pulmonary monitoring and early intervention to address pulmonary complications. Further research is warranted to explore the impact of hydroxyurea and the cumulative effect of vaso-occlusive events on lung function in SCD.

## Introduction

Initially described by James Herrick in an African-descent patient, the understanding of sickle cell disease (SCD) has come a long way [1]. Inherited as an autosomal recessive pattern, it occurs due to an abnormally formed hemoglobin, hemoglobin S (HbS), which results from the substitution of glutamic acid by valine at the sixth position of the beta-globin chain. The genotypes are sickle cell anemia (homozygous, HbSS), sickle beta-thalassemia (HbSβ), and sickle-hemoglobin C disease (HbSC), among others. Sickle cell trait is the carrier state with milder signs and symptoms [2]. India has the largest prevalence of SCD in South Asia, with central India accounting for the highest frequency of disease in the country, from South-Eastern Gujarat to South-Western Odisha.

Among the many complications of SCD, acute and chronic morbidity due to pulmonary involvement has been long established. It is also acknowledged to be the second most common cause of hospital admissions in sickle cell anemia (SCA), next only to vaso-occlusive crisis [3]. Pulmonary involvement in sickle cell syndromes occurs as acute chest syndrome (ACS), lower airway obstruction, airway hyper-responsiveness, and pulmonary hypertension (PAH). The extent of pulmonary involvement in SCD can be assessed by pulmonary function tests (PFT). The results of PFTs in children have shown varied results, with some studies indicating an obstructive pattern and others showing a restrictive type of abnormality. Even with the high burden of sickle cell cases in India, there are only a few studies on the PFT in SCD-affected children. Therefore, we conducted a study aimed at assessing the pulmonary function abnormalities in children with SCD and comparing these findings with children who have other forms of anemia.

## Materials and methods

A cross-sectional study was conducted over two years from July 2022 to June 2024 at the pediatrics and pediatric hemato-oncology department of Kalinga Institute of Medical Sciences (KIMS), Bhubaneswar, Odisha, India. Ethical approval was obtained from the Institutional Ethics Committee of KIMS (approval number: KIIT/KIMS/IEC/988/2022). A total of 126 children were included in the study: 63 children with SCD and 63 children with other forms of anemia. Inclusion criteria for children with SCA were asymptomatic children between six and 18 years of age, either known or newly diagnosed cases of sickle cell disease (by high-performance liquid chromatography (HPLC)). The other groups comprised children aged between six and 18 years with anemia diagnosed by a complete blood count. Children with SCD with ACS or vaso-occlusive crises in the preceding two months and children who were known cases of asthma or on bronchodilator therapy or had pneumonia at presentation were excluded. Participants’ data, such as sociodemographic information and anthropometric measurements (weight, height, and body mass index (BMI)), were taken. A general systemic examination was done to rule out any cardiac, respiratory, or musculoskeletal abnormalities. In children with SCD, age at diagnosis, history of vaso-occlusive crisis or ACS, and duration of usage of hydroxyurea were taken.

Risk factors assessed were age, gender, BMI, time since diagnosis of SCD, type of SCD (SCA/sickle-beta thalassemia (SCβ)), history of ACS, history of vaso-occlusive crisis, and duration of use of hydroxyurea. Tests done included a baseline complete blood count, sickling test, HPLC test for those who tested positive with the sickling test, and PFT via spirometry. Lung function assessment was done by spirometry test (Minispir II device) according to the American Thoracic Society (ATS) and European Respiratory Society (ERS) guidelines for children [4]. The parameters such as forced vital capacity (FVC), FVC%, forced expiratory volume in one second (FEV1), FEV1%, and FEV1/FVC were recorded. As children as young as six years were amongst the participants, a forceful expiratory blow of ≥3 seconds was considered acceptable, in accordance with the 2005 ATS guidelines [4]. The resulting data were analyzed to assess the type and extent of pulmonary function test abnormality prevalent in children with asymptomatic SCD as compared with children with other forms of anemia and were classified into normal, obstructive, restrictive, and mixed.

Statistical analysis

All continuous variables were expressed by mean ± SD and categorical variables by frequency and percentage after the normality assumption check by the Shapiro-Wilk test. Descriptive statistics were calculated for all the parameters. For comparison between case and control, Student's t-test was used. For measuring the association between categorical variables, chi-square test statistics were used. The type of anomaly compared with clinical and pulmonary parameters between case and control was done by a two-way ANOVA test. The statistical analysis was done by using IBM SPSS Statistics software, version 23.0 (IBM Corp., Armonk, NY). A p-value <0.05 was considered statistically significant.

## Results

Of the 126 children enrolled, the SCD group (N=63) comprised 38 boys (60.3%) and 25 girls (39.7%), and the control group (N=63) had 28 boys (44.4%) and 35 girls (55.6%). The mean age was slightly lower in children with SCD than those of controls (12.11 ± 2.79 and 13.65 ± 2.20 years, respectively). Anthropometric data showed that height and weight were lower in children with SCD, with the difference in height having a statistical significance (p=0.041). Body mass index was categorized as per WHO guidelines for boys and girls into severe thinness, thinness, normal, overweight, and obesity [5]. The average BMI in children with SCD was 16.20 ± 2.56 kg/m², while in the control group, it was 16.62 ± 2.27 kg/m², with no statistical significance. The anthropometric measurements and laboratory data are presented in Tables 1-2, respectively.

**Table 1:** Anthropometric data of the study participants SCD: sickle cell disease; BMI: body mass index

Anthropometric measurements	Study groups	Mean	Std. deviation	P-value
Age (years)	Children with SCD	12.11	2.79	0.001
	Control	13.65	2.20	
Height (m)	Children with SCD	1.42	0.16	0.041
	Control	1.47	0.10	
Weight (kg)	Children with SCD	33.84	10.93	0.143
	Control	36.33	7.82	
BMI (kg/m^2^)	Children with SCD	16.20	2.56	0.333
	Control	16.62	2.27	

**Table 2:** Laboratory parameters of the study group Hb: hemoglobin; SCD: sickle cell disease; WBC: white blood cell; RBC: red blood cell; MCV: mean corpuscular volume; MCH: mean corpuscular hemoglobin; MCHC: mean corpuscular hemoglobin concentration; RDW: red cell distribution width

Parameter	Reference ranges	Study groups	Mean	Std. deviation	P – value
Hb (gm/dL)	12.0 – 15.0	Children with SCD	9.12	1.82	<0.001
		Control	7.59	2.16	
WBC (10^3^/uL)	4.0 – 10.0	Children with SCD	8.955	4.091	<0.001
		Control	4.846	3.439	
RBC (10^6^/uL)	4.5 – 5.5	Children with SCD	3.43	1.04	0.529
		Control	3.31	1.15	
PLATELET (10^3^/uL)	150.0 – 410.0	Children with SCD	237.26	131.94	0.784
		Control	230.97	123.14	
PCV (%)	36.0 – 46.0	Children with SCD	28.01	5.29	0.007
		Control	24.74	7.84	
MCV (fL)	83.0 -101.0	Children with SCD	83.06	13.43	0.05
		Control	78.49	12.38	
MCH (pg)	27.0 – 32.0	Children with SCD	26.69	5.98	0.622
		Control	29.44	43.85	
MCHC (gm/dL)	31.5 – 34.5	Children with SCD	32.48	1.90	<0.001
		Control	30.59	2.52	
RDW (%)	11.6 – 14.0	Children with SCD	19.59	6.60	<0.001
		Control	25.38	8.56	

Clinical symptomatology was similar in both groups. Forty-four children with SCD (69.8%) and 40 children in controls (63.5%) had fatigue. Similarly, 44 children with SCD (69.8%) and 42 children in the control group (66.7%) complained of generalized weakness.

Anemia was classified as mild, moderate, or severe anemia as per WHO guidelines [6]. Mild anemia varied according to age, with the cut-off being 11.0 - 11.4 gm/dL in children between five and 11 years of age, 11.0 - 11.9 gm/dL in children between 12 and 14 years of age and non-pregnant girls, 11.0 - 11.9 gm/dL in non-pregnant girls between the ages of 15 and 18 years, and 11.0 - 12.9 gm/dL in boys of 15 to 18 years of age. Moderate anemia had a cut-off between 8.0 - 10.9 g/dL, while severe anemia was less than 8.0 g/dL in all age groups and genders.

As per the classification, 40 children with SCD (63%) had moderate anemia, whereas 36 children in controls (57%) had severe anemia. Complete blood count parameters were compared between cases and controls, and statistical significance was found for hemoglobin, white blood cell count (WBC), packed cell volume (PCV), mean corpuscular volume (MCV), mean corpuscular hemoglobin concentration (MCHC), and red cell distribution width (RDW). The mean hemoglobin of children with SCD was 9.12 ± 1.82 gm/dL, while in controls it was 7.59 ± 2.16 gm/dL with statistical significance (p < 0.001). It was observed that mean hemoglobin was lower in controls as compared to cases. Amongst other red cell indices, RDW was also found to be higher in controls than in children with SCD (25.38 ± 8.56 and 19.59 ± 6.60, respectively, p < 0.001). Both MCV and MCHC, however, were higher in children with SCD than in controls, with statistical significance (p = 0.05 and p < 0.001, respectively). We had two subgroups within sickle cell disease: the SCA group with 52 children (83%) and the SCβ group with 11 children (17%).

The mean value of FVC in children with SCD was 1.96 ± 0.61 L, which was significantly lower than the value in controls, which was 2.32 ± 0.60 L (p = 0.001). Similarly, the mean values of FEV1 were also significantly lower in children with SCD than in the control group (1.64 ± 0.60 L and 1.91 ± 0.54 L, respectively) with p = 0.011. A higher ratio of FEV1/FVC was seen in children with SCD than in controls, although not statistically significant. Normal spirometry pattern was found in only 17 children with SCD (26.9%) and in 27 (42.8%) of controls. Abnormal PFT was seen in both groups. The predominant pattern was the restrictive type in both groups, seen with 40 children with SCD (63.4%) and 25 (39.6%) controls. Obstructive pattern was seen in four children with SCD (6.3%) and nine (14.2%) controls, which was not statistically significant. The comparisons of PFT of both groups are depicted in Table 3, and the pattern of spirometry in both study groups has been shown in Table 4.

**Table 3:** Comparison of PFT parameters in both groups PFT: pulmonary function test; FVC: forced vital capacity; FEV1: forced expiratory volume in one second; SCD: sickle cell disease

PFT parameters	Study group	Mean	Std. deviation	P-value
FVC (L)	Children with SCD	1.96	0.61	0.001
	Controls	2.32	0.60	
FVC%	Children with SCD	75.33	15.54	0.041
	Controls	80.84	14.35	
FEV1 (L)	Children with SCD	1.65	0.60	0.011
	Controls	1.91	0.54	
FEV1%	Children with SCD	75.68	16.01	0.611
	Controls	77.22	17.80	
FEV1/FVC	Children with SCD	86.31	13.16	0.108
	Controls	82.75	11.34	

**Table 4:** Pattern of spirometry in both study groups SCD: sickle cell disease

Pattern of spirometry	Study group	N (%)	P-value
Normal	Children with SCD	17 (26.9%)	0.264
	Controls	27 (42.8%)	
Restrictive	Children with SCD	40 (63.4%)	
	Controls	25 (39.6%)	
Obstructive	Children with SCD	4 (6.3%)	
	Controls	9 (14.2%)	
Mixed	Children with SCD	2 (3.17%)	
	Controls	2 (3.17%)	

**Figure 1:**
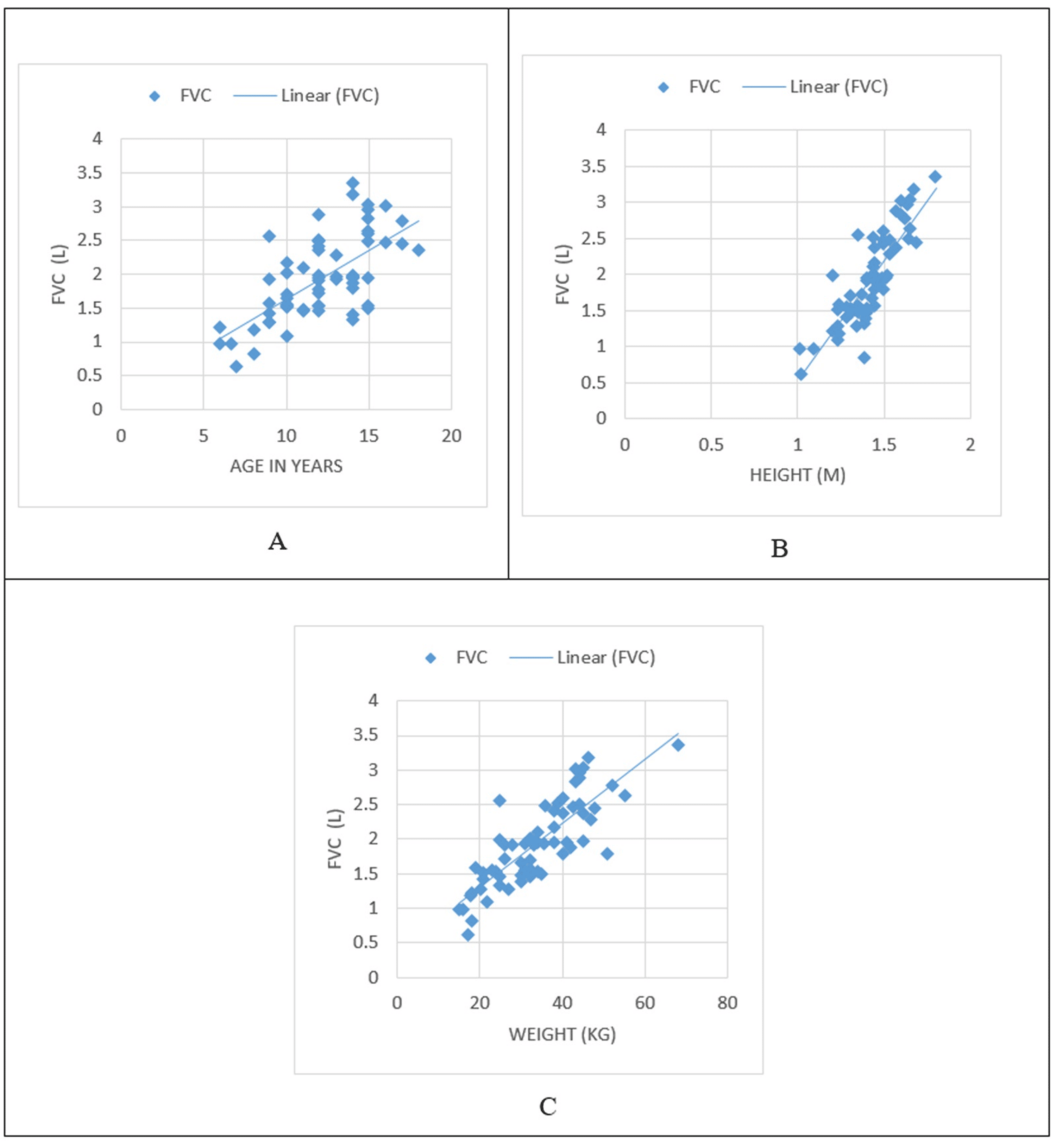
Correlation of FVC with age and anthropometric parameters A: Correlation of FVC with age; B: Correlation of FVC with height; C: Correlation of FVC with weight FVC: forced vital capacity

**Figure 2:**
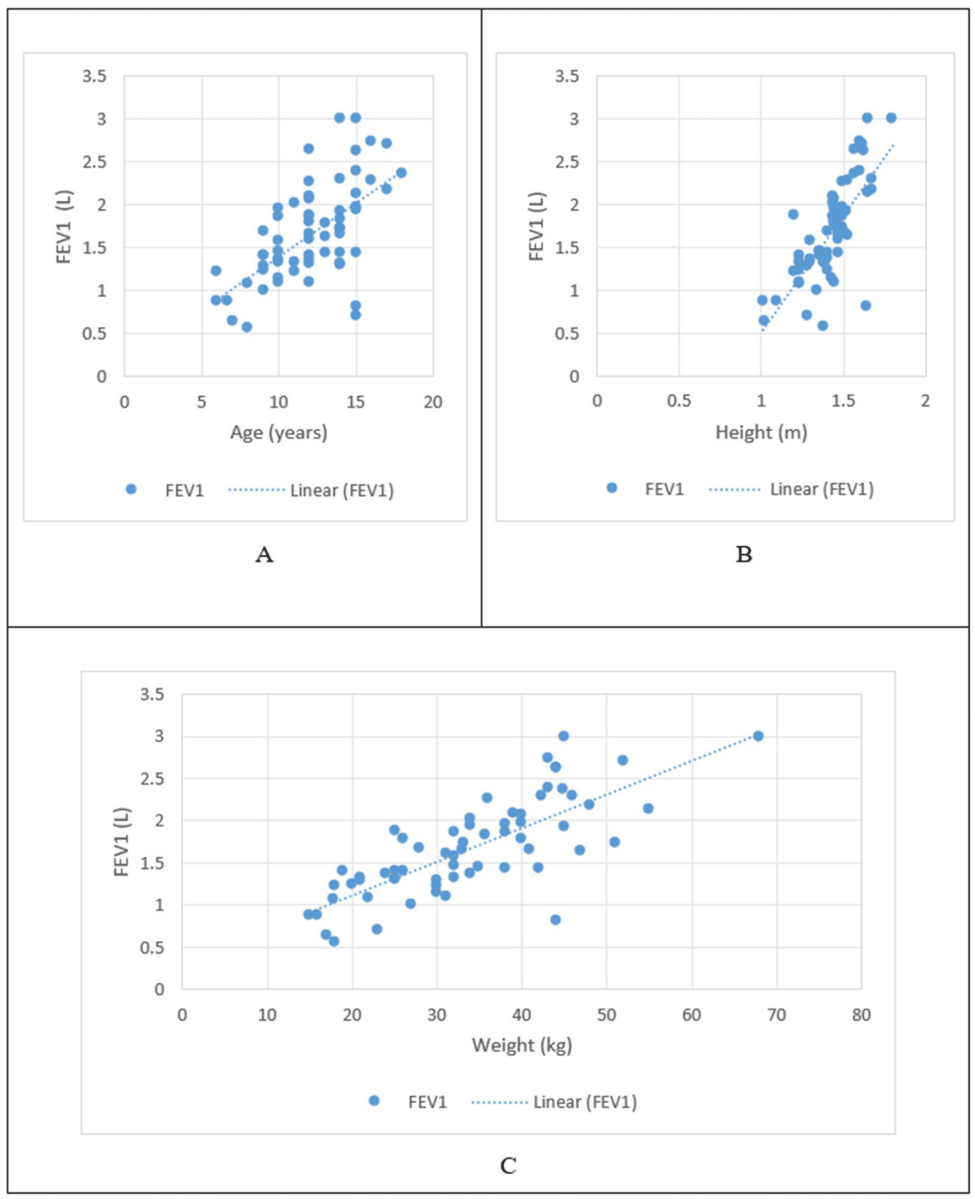
Correlation of FEV1 with age and anthropometric parameters A: Correlation of FEV1 with age; B: Correlation of FEV1 with height; C: Correlation of FEV1 with weight FEV1: forced expiratory volume in one second

In children with SCD, the PFT parameters of FVC and FEV1 both had a positive correlation with age, height, and weight, as shown in Figures 1-2, respectively.

Various risk factors were assessed in children with SCD, including age, gender, previous episodes of vaso-occlusive crises and ACS, history of blood transfusion, treatment with hydroxyurea, and genotype of SCD, which are tabulated in Table 5.

**Table 5:** Assessment of risk factors on PFT outcomes in children with SCD F: female; M: male; VOC: vaso-occlusive crisis; ACS: acute chest syndrome; SCA: sickle cell anemia; SCβ: sickle cell beta-thalassemia; HbSS: homozygous sickle cell anemia; HbSβ: sickle beta-thalassemia

Risk factors		Normal	Restriction	Obstruction	Mixed	P-value
Age group (years)	6-9	2 (16.7%)	7 (58.3%)	2 (16.7%)	1 (8.3%)	0.012
	10-13	13(48.1%)	13 (48.1%)	0	1 (3.7%)	
	14-18	2 (8.3%)	20(83.3%)	2 (8.3%)	0	
Sex	F	8 (3.2%)	16 (64%)	0	1 (4%)	0.375
	M	9 (23.7%)	24 (63.2%)	4 (10.5%)	1 (2.6%)	
VOC	No	0	4 (80%)	1 (20%)	0	0.325
	Yes	17 (29.3%)	36 (62.1%)	3 (5.2%)	2 (3.4%)	
ACS	No	5 (18.5%)	20 (74.1%)	1 (3.7%)	1 (3.7%)	0.449
	Yes	12 (33.3%)	20 (55.6%)	3 (8.3%)	1 (2.8%)	
History of blood transfusions	No	7 (30.4%)	14 (60.9%)	2 (8.7%)	0	0.645
	Yes	10 (25%)	26 (65%)	2 (5%)	2 (5%)	
Severity of anemia	Normal	0	2 (100%)	0	0	0.112
	Mild	4 (66.7%)	2 (33.3%)	0	0	
	Moderate	12 (33.3%)	22 (56.4%)	2 (5.1%)	2 (5.1%)	
	Severe	0	13 (86.7%)	2 (13.3%)	0	
Hydroxyurea	Never had hydroxyurea	2 (14.3%)	9 (64.3%)	2 (14.3%)	1 (7.1%)	0.289
	On hydroxyurea	15 (30.6%)	31 (63.3%)	2 (4.1%)	1 (2%)	
Genotype	SCA (HbSS)	16 (30.8%)	33 (63.5%)	2 (3.8%)	1 (1.9%)	0.112
	SCβ (HbSβ)	1 (9.1%)	7 (63.6%)	2 (18.2%)	1 (9.1%)	

The age group was found to be statistically significant (p = 0.012), with restrictive patterns being more common in all age groups. Of the children in the 14-18 age group (n=24), 20 children (83.3%) had a restrictive pattern. The obstructive pattern of PFT was seen in two children (16.7%) in the age group of six to nine years and in two children (8.3%) in the age group of 14-18 years. Gender did not have a statistical significance in the pattern of abnormality in children with SCD. Fifty-eight children (92.8%) had at least one previous episode of vaso-occlusive crisis, and 36 children (57.1%) had at least one episode of ACS. Amongst the children with these factors and those with a history of blood transfusion (n=40 children), the restrictive pattern was the most common abnormality, rendering clinical importance but with no statistical significance. Of the 49 children on regular treatment with hydroxyurea, 31 children (63.3%) had a restrictive pattern of PFT abnormality, with no statistical significance. Of the 63 children with SCD, 52 children (83%) had HbSS and 11 children (17%) had HbSβ. The genotype had no statistical significance in the type of abnormality of PFT, although the restrictive pattern was more common in both genotypes.

## Discussion

This study presents a comprehensive analysis of clinicodemographic, laboratory, and PFT findings in children with SCD compared to controls. The findings illustrate a complex interaction of hematological, anthropometric, and pulmonary function differences, with specific trends among children with SCD.

The study reveals that children with SCD had lower height and weight measurements compared to controls, aligning with established literature that describes growth retardation as common in SCD due to chronic anemia, increased metabolic demands, and nutritional deficits. The statistical significance in height, albeit slight, reinforces the impact of SCD on physical growth, potentially due to lower oxygen-carrying capacity and recurrent infections. The BMI values, however, did not significantly differ between groups, suggesting that while SCD impacts overall growth, it may not affect body composition (as BMI was categorized per WHO guidelines). There are contrasting studies that showed similar age and gender distribution but with statistically significant low BMI and hemoglobin levels in cases [7].

Laboratory findings indicated that children with SCD had significantly higher hemoglobin levels compared to controls, though both groups exhibited low levels, reflecting the prevalence of anemia. The increased hemoglobin in SCD patients compared to controls, likely due to compensatory mechanisms in chronic hemolytic anemia, highlights the body’s attempt to counterbalance chronic oxygen deprivation. Additionally, RDW was significantly higher in controls, suggesting greater variability in red cell size possibly due to the diversity of anemic etiologies in this group. The values of WBC, PCV, MCV, and MCHC were significantly different between groups, illustrating hematologic distinctions tied to SCD. Notably, children with SCD had a higher MCV and MCHC as compared to controls, reflecting the hemolytic and microcytic nature of sickle cells.

Significant differences in pulmonary function were observed, with children with SCD demonstrating lower mean values of FVC and FEV1 compared to controls. The reduced FVC and FEV1 in children with SCD indicate restricted lung volumes, aligning with previous studies suggesting restrictive lung disease in this population. Interestingly, while the FEV1/FVC ratio was higher in children with SCD, this finding was not statistically significant. There are similar studies that indicate that children with SCD had significantly lower FEV1 and FVC than controls matched for sex, race, and height, but had similar FEV1/FVC. A study conducted on Indian children showed significantly lower FEV1, and FVC in the study group (HbSS cases) compared to normal healthy controls [8-10].

The restrictive pattern was predominant in both groups but was markedly higher in children with SCD (n=40, 63.4%) as compared to controls (n=25, 39.6%). Restrictive patterns, common in SCD, may arise from chronic inflammation, fibrosis, or chest wall deformities. The study shows that only 17 children with SCD (26.9%) had normal spirometry results, significantly lower than in the control group, which had 27 children (42.8%). This disparity in lung function reinforces the need for regular monitoring and potential early intervention in SCD patients to manage pulmonary complications. Though obstructive patterns were seen in a small subset of SCD cases, the findings suggest that restrictive defects are the primary PFT abnormality, aligning with SCD’s clinical profile. Our study had a good correlation with that of Purohit et al [11], in which a restrictive pattern was more common, but hemoglobin was significantly low in SCD compared to healthy controls. In contrast, an obstructive pattern was found to be more common in one study attributed to recurrent chest infections and acute chest syndrome in those patients [12].

Several risk factors, including age, history of vaso-occlusive crises, ACS, and blood transfusions, were examined for their influence on PFT abnormalities in children with SCD. A higher prevalence of restrictive abnormalities was seen in older children, particularly those aged 14-18 years. The correlation between age and restrictive patterns could be indicative of cumulative pulmonary injury due to recurrent vaso-occlusive events and chronic inflammation. Episodes of vaso-occlusive crises and ACS were notably common in SCD children (92.8% and 57.1%, respectively), though they did not reach statistical significance with PFT abnormalities. Nevertheless, these factors are clinically relevant as they increase the risk of lung tissue damage and promote the restrictive pattern seen in spirometry. However, in a study that reviewed the Pulmonary Hypertension and Hypoxic (PUSH) Response in SCD, it was seen that an obstructive pattern was more common due to the concurrence of asthma. In our study, only four children (6.3%) with SCD had an obstructive pattern, and they had a history of acute chest syndrome and vaso-occlusive crisis in the past [13].

Among children receiving hydroxyurea, a notable proportion (n=31, 63.3%) exhibited restrictive PFT abnormalities, though without statistical significance. Hydroxyurea, a treatment used to reduce vaso-occlusive events and enhance hemoglobin F production, may have a protective effect on lung function, though further research is needed to confirm its impact on PFT outcomes in SCD. Similar findings were reported in some studies where there was no statistically significant difference in the parameters of PFTs of patients on hydroxyurea [14,15].

Additionally, the presence of different genotypes within the SCD group, specifically HbSS and HbSβ, was noted, but genotype did not significantly affect the type of PFT abnormality observed. Our study also documented that higher HbF levels resulted in a normal spirometry result, hence having a protective role. Our analysis also showed that hemoglobin F% had a positive correlation with FVC and a negative correlation with FEV1 and FEV1/FVC, but there was no statistical significance. In contrast to our study, there was a mild to moderate negative correlation with FVC, FEV1, FVC%, FEV1%, and FEV1/FVC with HbF, with no statistical significance as observed by Aglave et al. [16].

While our study offered valuable insights into pulmonary function among children with SCD, it was limited to a single center, which may affect the generalizability of these findings to a broader population. Spirometry in this study was based on the Knudson reference equations; however, using the Global Lung Initiative (GLI) 2012 reference equations might have yielded more accurate results, as they account more precisely for age, height, and ethnicity [17].

We recommend early and periodic spirometry screening in children with SCD from age six onward to facilitate early detection and intervention. Children with SCD who exhibit respiratory symptoms or have a history of ACS should particularly undergo pulmonary function testing. Regular respiratory exercises and the use of a three-ball incentive spirometer may aid in maintaining lung function and potentially prevent further deterioration. Future research, ideally through multi-center studies, would be beneficial to validate these findings and establish ethnically relevant reference ranges tailored to the Indian pediatric population.

## Conclusions

This study underscores that children with SCD exhibited significant clinicodemographic, hematologic, and pulmonary disparities compared to those in the control group, particularly with reduced lung function as indicated by restrictive PFT patterns. The frequent restrictive defects in children with SCD necessitate ongoing pulmonary assessments to mitigate long-term respiratory complications. Ensuring that pulmonary function is tested at regular intervals in children with SCD results in early detection of lung function abnormality; hence, allowing for early initiation of incentive spirometry and chest physiotherapy. These findings highlight the multifaceted impacts of SCD on pediatric health, warranting further exploration of tailored interventions to improve both pulmonary outcomes and overall quality of life in these children.
